# Risk factors for bad outcome in pediatric epidural hematomas: a systemic review

**DOI:** 10.1186/s41016-019-0167-6

**Published:** 2019-08-08

**Authors:** Peter Spazzapan, Klemen Krašovec, Tomaž Velnar

**Affiliations:** Unit of Pediatric Neurosurgery, Department of Neurosurgery, University Medical Centre Ljubljana, Zaloška 7, 1000 Ljubljana, Slovenia

**Keywords:** Head trauma, Hemiparesis, Glasgow coma scale, Craniotomy, Rehabilitation

## Abstract

**Background:**

Pediatric epidural hematomas (EDH) represent a neurosurgical emergency. Both surgical and conservative treatment can lead to a good clinical outcome. The aim of the study was to review our series of pediatric EDH and to determine the clinical and radiologic factors, which can influence the final outcome.

**Methods:**

All children aged from 0 to 16 that have been treated between 2013 and 2017 for cranial EDH have been selected.

**Results:**

Thirty children have been included in the study. Seventeen cases have been treated with surgical evacuation and 13 conservatively. Six months after the trauma, the outcome was excellent (mRS 0) in 25/30 (83.3%) cases, mild deficits (mRS 1–2) were present in 4/30 (13.3%), and severe deficits (mRS 3–5) in 1/30 (3.3%) cases. Only a GCS (Glasgow Coma Scale) below 8 at admission was significantly related to the presence of a neurologic deficit at 6 months (*p* = 0.048).

**Conclusions:**

EDH can be managed with excellent outcomes. Even in the presence of bad initial clinical and radiologic conditions, a correct treatment strategy can lead to a good recovery. In our series, only a GCS below 8 at admission was significantly related to the presence of neurological sequelae.

## Background

Epidural hematomas (EDH) account for about 2–3% of all head injuries in the pediatric population and represent 1–6% of all diagnoses in children hospitalized after traumatic brain injury [1-6]. They are known as an important neurosurgical emergency, which has to be diagnosed rapidly and treated adequately. The treatment of EDH can be surgical or conservative, and the indication for surgery is based on clinical and radiological parameters. The availability of an advanced pediatric trauma care and urgent surgery can lead to very good treatment outcome.

This article presents a series of pediatric EDH treated both conservatively and surgically at the Pediatric Neurosurgical Unit of the University Medical Centre Ljubljana. The aim of the study was to review the clinical and radiological aspects in these patients and to determine which of them may influence the outcome.

## Methods

A single-center, retrospective study was performed in order to evaluate the clinical, radiological, and surgical results of all children admitted to the University Medical Centre Ljubljana with the diagnosis of EDH between February 2013 and October 2017. The inclusion criteria were (a) the pediatric age (below 16 years) and (b) the presence of a cranial EDH among admission or discharge diagnoses.

Clinical charts and radiological exams were reviewed and included the following parameters: (a) age, (b) sex, (c) mechanisms of injury, (d) Glasgow Coma Scale (GCS) at admission, (e) abnormal pupillary response, (f) thickness and location of the EDH, (g) presence of midline shift, (h) associated cranial fracture, (i) treatment strategy, (j) time to eventual surgical treatment, and (k) duration of hospital stay. The outcome was assessed according to the modified Rankin Scale (mRS): 0 in case of no symptoms at all, 1 to 2 in case of slight disability, and 3 to 5 in case of moderate to severe disability. A score of 6 was designated in case of death.

All children have been admitted to the trauma center. In case of GCS score below 8 and in case of respiratory or cardiovascular compromise, they have been treated according to the advanced trauma life support protocol. Our protocol considered to perform a cranial CT scan in case of (a) post-traumatic seizures, (b) decline of consciousness (GCS below 14 or below 15 2 h after trauma), (c) suspect of depressed skull fracture, (d) occurrence of CSF leak, (e) presence of neurological deficits, and (f) anticoagulant treatment.

The indication for surgery was based on the neurological condition (GCS) and on radiologic findings (EDH thickness, midline shift, associated dislocated skull fracture). Some children have been operated urgently, within 4 h from admission, while others more than 12 h after trauma. All children, who underwent the operative treatment, received the same surgical procedure, which involved a standard craniotomy, EDH evacuation, application of dural tenting sutures, application of an epidural drain, and bone flap fixation with resorptive sutures.

In cases where sedation and ventilation were needed postoperatively, an intracranial pressure (ICP) sensor was inserted and the values were monitored in the pediatric intensive care unit (ICU).

Independently from the received treatment, a control CT scan or MRI was done in all patients 12 to 24 h after the first examination.

All children were hospitalized in the pediatric ICU until the clinical condition began to stabilize. When the acute phase of treatment was concluded, they were discharged home or to the rehabilitation unit.

The mRS was assessed at the discharge from the hospital and after 6 months. Statistical analysis was done with the SPSS software version 18.0 (SPSS Inc., Chicago, IL, USA). A binary logistic regression analysis was performed to define the prognostic influence of single variables. Variables included in the analysis were (a) GCS at admission, (b) EDH size, (c) unilateral fixed pupil, (d) associated intracranial lesions, and (e) type of treatment. The significance level was *p* < 0.05.

## Results

Between February 2013 and October 2017, 30 pediatric patients with the diagnosis of EDH were admitted. Among the patients, 5/30 (16.7%) were girls and 25/30 (83.3%) were boys (Table 1). The incidence was 0.52 cases per month. The average age at the admission was 6.7 years (min. 6 months, max. 16 years). Three out of thirty (10%) children were younger than 1 year, 8/30 (26.7%) children were between 1 and 4 years of age, 11/30 (36.7%) were between 5 and 10 years of age, and 8/30 (26.7%) were between 10 and 16 years of age (Table 1). The mean follow-up was 42.5 months (min. 12 months, max. 69 months).

**Table 1:** General data of the pediatric population included in the study

	All cases	Separate groups	
		Operated group	Not operated group
Male patients	25/30 (83.3%)	16/17 (94.1%)	9/13 (69.2%)
Female patients	5/30 (16.7%)	1/17 (5.9%)	4/13 (30.8%)
Median age	6.7 years	5.9 years	7.6 years
< 1 year	3/30 (10%)	3/17 (17.6%)	0/13 (0%)
1–4 years	8/30 (26.7%)	5/17 (29.4%)	3/13 (23.1%)
5–10 years	11/30 (36.7%)	4/17 (23.5%)	7/13 (53.8%)
10–16 years	8/30 (26.7%)	5/17 (29.4%)	3/13 (23.1%)

In 27/30 (90%) cases, the cause of EDH was a fall, which occurred most frequently in domestic environment or in accidents involving a bicycle or a skateboard. In 3/30 (10%) cases, the cause was a motor vehicle accident.

The GCS at the admission to the emergency department was 14 to 15 in 20/30 (66.7%) cases, 9 to 13 in 3/30 (10%) cases, and below 8 GCS in 7/30 (23.3%) cases (Table 2). The average GCS at admission was 12.5.

**Table 2:** Clinical conditions at admission (GCS and mydriasis) and deterioration of the level of consciousness during clinical observation

	All cases	Separate groups	
		Operated group	Not operated group
14–15 GCS	20/30 (66.7%)	9/17 (52.9%)	11/13 (84.6%)
9–13 GCS	3/30 (10%)	2/17 (11.8%)	1/13 (7.7%)
< 8 GCS	7/30 (23.3%)	6/17 (35.3%)	1/13 (7.7%)
Median GCS at admission	12.5	11.7	13.6
Unilateral fixed pupil	4/30 (13.3%)	3/17 (17.6%)	1/13 (7.7%)
Early and late clinical deterioration	5/30 (16.7%)	4/17 (23.5%)	1/13 (7.7%)

**Fig. 1:**
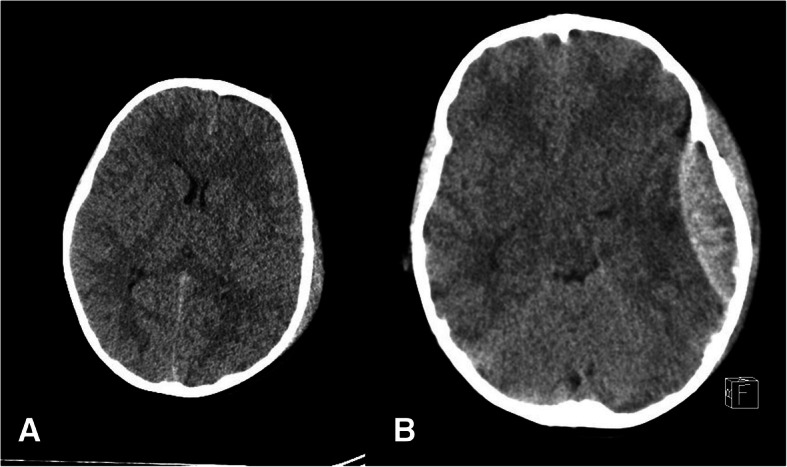
CT scan at admission (**a**) and 3 h after admission (**b**) of children presenting with early clinical deterioration and a typical lucidity interval

**Fig. 2:**
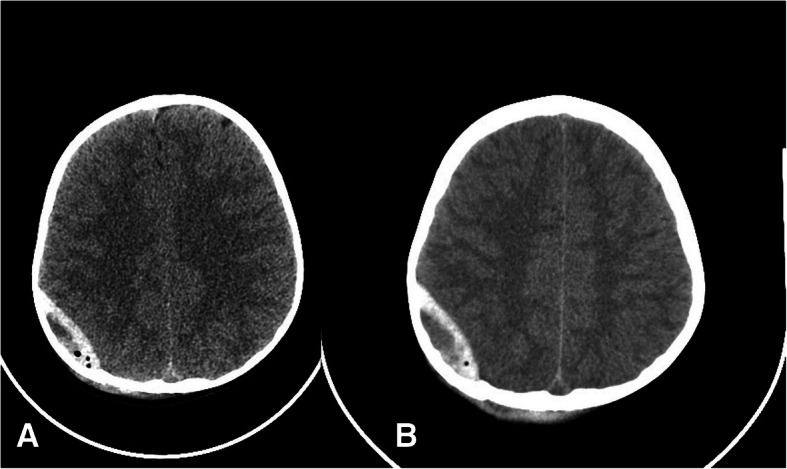
CT scan at admission (**a**) and 3 days after admission (**b**) of a child presenting a late clinical deterioration. The EDH was surgically evacuated

During the observation, a deterioration of the level of consciousness was observed in 5/30 (16.7%) patients. In 3 of them, the clinical condition has worsened early, with a typical lucidity interval (Fig. 1), while in 2 cases, the deterioration occurred later, during the hospitalization (Fig. 2). In 4/30 (13.3%) children, a unilateral fixed pupil due to the transtentorial herniation was observed.

**Fig. 3:**
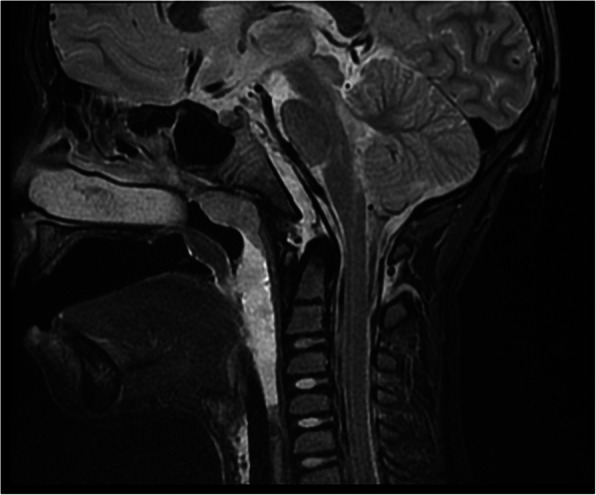
A retroclival EDH treated conservatively. Six months after trauma, the child still presented a bilateral 6th nerve palsy

In all patients, the primary diagnostic modality was a CT scan. The location of the EDH was as follows (Table 3): 11/30 (36.7%) were parietotemporal, 9/30 (30%) were temporal, 8/30 (26.7%) were frontotemporal, 1/30 (3.3%) was occipital, and 1/30 (3.3%) was retroclival (Fig. 3). The mean size of EDH was 14.6 mm (min. 4 mm, max. 40 mm), and a midline shift was present in 15/30 (50%) cases.

**Table 3:** Radiologic data of the EDH included in the study

		All cases	Separate groups	
			Operated group	Not operated group
EDH location	Parietotemporal	11/30 (36.7%)	9/17 (52.9%)	2/13 (15.4%)
	Temporal	9/30 (30%)	4/17 (23.5%)	5/13 (38.5%)
	Frontotemporal	8/30 (26.7%)	4/17 (23.5%)	4/13 (30.8%)
	Occipital	1/30 (3.3%)	0/17 (0%)	1/13 (7.7%)
	Retroclival	1/30 (3.3%)	0/17 (0%)	1/13 (7.7%)
Mean EDH thickness		14.6 mm	20.5 mm	6.8 mm
Midline shift		15/30 (50%)	15/17 (88.2%)	0/13 (0%)

**Fig. 4:**
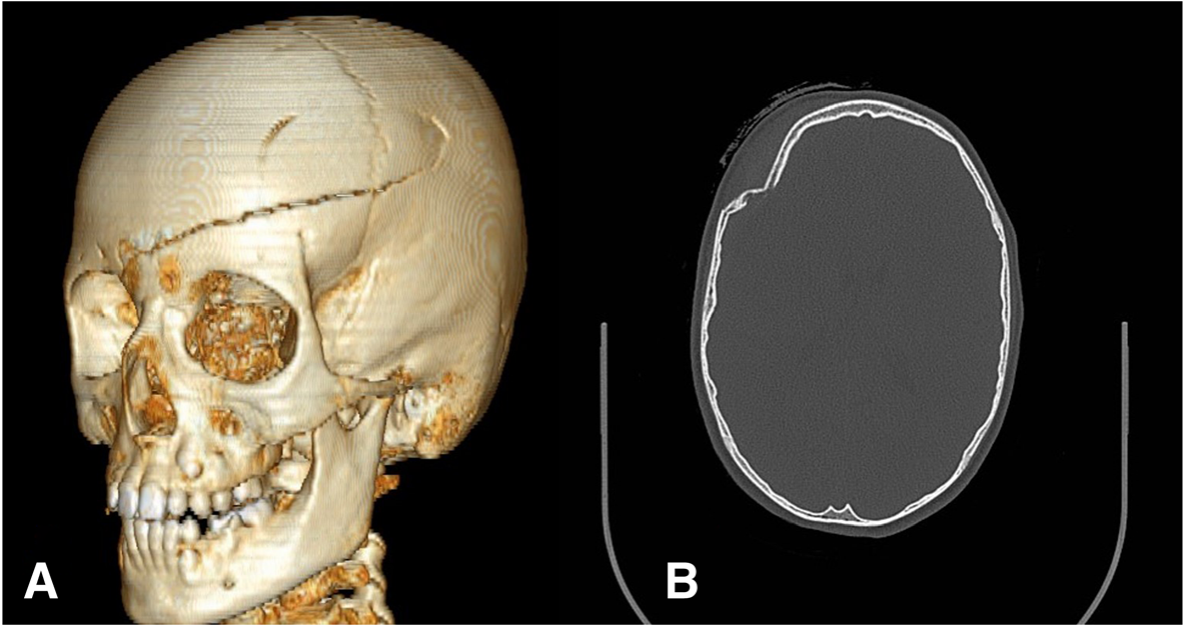
A linear skull fracture (**a**) and a depressed skull fracture (**b**), which needed surgical repair

A skull fracture was present in 21/30 (70%) patients (Fig. 4): in 19/21 (90.5%) patients, the fracture was located at the cranial vault, in 2/21 (9.5%) at the skull base, in 1/21 (4.8%) at the zygomatic arch, and in 1/21 (4.8%) at the orbital floor. Five out of thirty (16.7%) patients had a dislocated fracture of the cranial vault (Table 4).

**Table 4:** Associated cranial fractures and associated intracranial lesions

		All cases	Separate groups	
			Operated group	Not operated group
Skull fracture		21/30 (70%)	11/17 (64.7%)	10/13 (76.9%)
Dislocated fracture		5/30 (16.7%)	5/17 (29.4%)	0/13 (0%)
Associated intracranial lesion		13/30 (43.3%)	8/17 (47%)	5/13 (38.4%)
	Contusion	6/30 (20%)	4/17 (23.5%)	2/13 (15.4%)
	SDH	3/30 (10%)	2/17 (11.8%)	1/13 (7.7%)
	SAH	3/30 (10%)	1/17 (5.9%)	2/13 (15.4%)
	Brain edema	5/30 (16.7%)	2/17 (11.8%)	3/13 (23.1%)
	CVI	1/30 (3.3%)	1/17 (5.9%)	0/13 (0%)
	Carotid dissection	1/30 (3.3%)	1/17 (5.9%)	0/13 (0%)

In 17/30 (56.7%) cases, the EDH represented an isolated intracranial injury, while 13/30 (43.3%) patients had at least one associated intracranial lesion. The preoperative CT scan detected a contusion in 6/30 (20%) cases, a subdural hematoma (SDH) in 3/30 (10%), a subarachnoid hemorrhage (SAH) in 3/30 (10%), and brain edema in 1/30 (3.3%) cases. The control CT scan or MRI showed brain edema in 4/30 (13.3%) cases, a cerebrovascular insult (CVI) in 1/30 (3.3%) cases, and a dissection of the internal carotid artery in 1/30 (3.3%) cases.

No seizures were documented neither in the acute phase of treatment, nor during follow-up.

The average length of hospitalization in the ICU was 2.8 days, while the average length of hospitalization for children with EDH was 9.6 days (min. 1 day, max. 30 days) (Table 5).

**Table 5:** Time of ICU hospitalization and total hospital stay of the children included in the study

	All cases	Separate groups	
		Operated group	Not operated group
ICU hospitalization	2.8 days	3.5 days	0.8 days
Total hospital stay	9.6 days	11.2 days	7.7 days

The overall outcome at the discharge from the hospital was as follows (Table 6): mRS 0 in 23/30 (76.7%) patients, mRS 1–2 in 6/30 (20%), and mRS 3–5 in 1/30 (3.3%) patients. There was no mortality (mRS 6).

**Table 6:** Clinical data (mRS) at the discharge from the hospital and 6 months after the discharge from hospital

		All cases	Separate groups	
			Operated group	Not operated group
mRS at discharge	mRS 0	23/30 (76.7%)	13/17 (76.4%)	10/13 (76.9%)
	mRS 1–2	6/30 (20%)	3/17 (17.6%)	3/13 (23, 1%)
	mRS 3–5	1/30 (3.3%)	1/17 (5.9%)	0/13 (0%)
mRS 6 months after discharge	mRS 0	25/30 (83.3%)	13/17 (76.4%)	12/13 (92.3%)
	mRS 1–2	4/30 (13.3%)	3/17 (17.6%)	1/13 (7.7%)
	mRS 3–5	1/30 (3.3%)	1/17 (5.9%)	0/13 (0%)

**Fig. 5:**
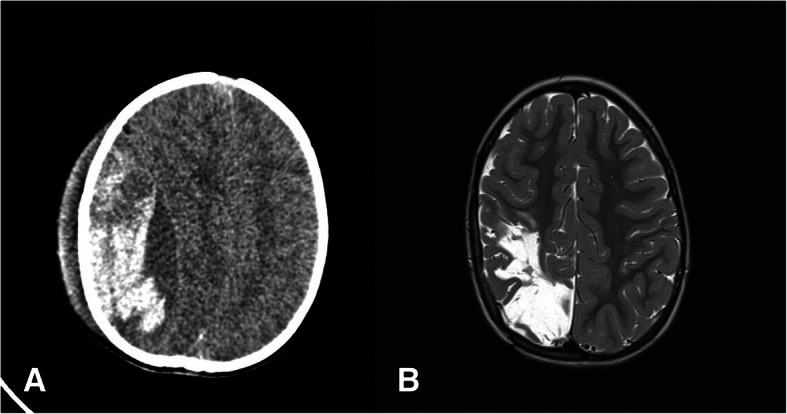
CT scan of the child with a large EDH (**a**) and the MRI performed 2 years after the trauma (**b**) showing an ischemic area in the territory of the right middle cerebral artery. Six months after surgery, the child still presented a severe hemiparesis, which resolved almost completely 3 years after the trauma

Six months after the trauma, the outcome was the following: mRS 0 in 25/30 (83.3%) cases, mRS 1–2 in 4/30 (13.3%) cases, and mRS 3–5 in 1/30 (3.3%) cases. Among the patients classified as mRS 1–2, we observed a mild hemiparesis in 3 cases, a mild mental disturbance in 3 cases, and a bilateral 6th nerve palsy in one case, which occurred in the child with the retroclival EDH. The child classified as mRS 3–5 suffered from a severe hemiparesis due to a postoperative ischemic stroke in the middle cerebral artery territory (Fig. 5). The hemiparesis improved moderately 6 months after the trauma, and after 3 years, an almost complete recovery was observed.

The statistical analysis was performed to assess which parameters were significantly related to the outcome. Only a GCS below 8 at admission was significantly related to a bad outcome (mRS 1–5) (*p* = 0.048).

EDH size (*p* = 0.298), unilateral fixed pupil (*p* = 0.646), associated intracranial lesions (*p* = 0.568), and type of treatment (*p* = 0.231) were not significantly related to a bad outcome.

### Operated group of patients

**Fig. 6:**
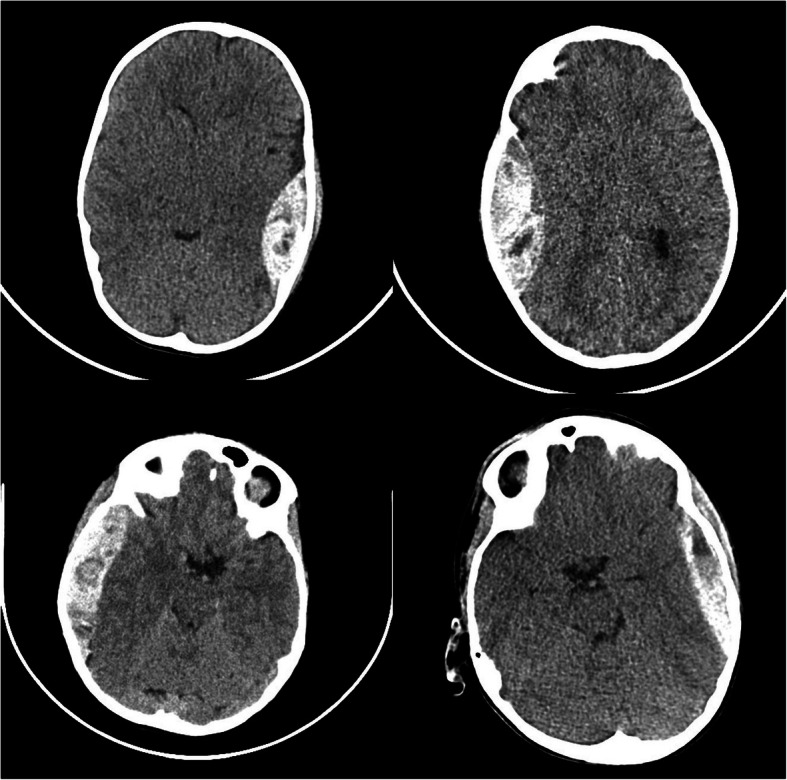
CT scans of 4 distinct cases of surgically treated EDH

Seventeen out of thirty (56.7%) children with EDH have been treated surgically (Fig. 6). The average age in the operated group was 5.9 years (min. 6 months, max. 16 years). There were 16/17 (94.1%) boys and 1/17 (5.9%) girl. Among the operated children, 3/17 (17.6%) were younger than 1 year, 5/17 (29.4%) were between 1 and 4 years of age, 4/17 (23.5%) were between 5 and 10 years of age, and 5/17 (29.4%) were between 10 and 16 years of age (Table 1).

The GCS at the admission in the emergency department was 14 to 15 in 9/17 (52.9%) cases, 9 to 13 GCS in 2/17 (11.8%) cases, and below 8 GCS in 6/17 (35.3%) cases (Table 2). The average GCS at the admission among the operated children was 11.7.

Three out of seventeen (17.6%) operated children had preoperatively a unilateral fixed pupil. The deterioration of the level of consciousness during the observation occurred in 4/17 (23.5%) cases. In 3 of them, the time from trauma to the clinical deterioration was short (< 4 h) and it was related to an active bleeding and to EDH enlargement (Fig. 1). In 1 case, the deterioration occurred after 3 days, and it was mainly related to a slight enlargement of the EDH and to a prolonged mass effect (Fig. 2). This was the only child that needed a delayed operation, while 16/17 (94.1%) children have been operated early, within 4 h from admission.

The EDH was parietotemporal in 9/17 (52.9%) cases, frontotemporal in 4/17 (23.5%) cases, and temporal in 4/17 (23.5%) cases (Table 3). The mean size of EDH in the operated group of patients was 20.5 mm (min. 9 mm, max. 40 mm), and a midline shift was present in 15/17 (88.2%) patients.

A skull fracture was detected in 11/17 (64.7%) cases, and 5/17 (29.4%) fractures were dislocated (Table 4). Among the operated children, the EDH was associated with another intracranial lesion in 8/17 (47%) cases. The initial CT scan showed contusions in 4/17 (23.5%) patients, a SDH in 2/17 (11.8%) patients, and a SAH in 1/17 (5.9%) patients. The control CT or MRI showed brain edema in 2/17 (11.8%) cases, a CVI in 1/17 (5.9%) cases, and a carotid artery dissection in 1/17 (5.9%) cases.

The average length of the ICU stay was 3.5 days, and the average length of hospitalization among the operated children was 11.2 days (min. 4 days, max. 30 days) (Table 5).

The outcome at the discharge from the hospital was as follows: mRS 0 in 13/17 (76.4%) cases, mRS 1–2 in 3/17 (17.6%) cases (2 cases of cognitive impairment and 1 case of hemiparesis), and mRS 3–5 in 1/17 (5.9%) cases. Six months after the trauma, the outcome did not differ (Table 6).

Among the operated children, no mortality or surgery-related morbidity was observed. There was no EDH recurrence and no need for surgical revision.

### Not operated group of patients

Thirteen out of thirty (43.3%) cases of EDH have been treated conservatively. The average age within this group was 7.6 years (min. 3 years, max. 12 years).

There were 4/13 (30.8%) girls and 9/13 (69.2%) boys. Three out of thirteen (23.1%) children were between 1 and 4 years of age, 7/13 (53.8%) between 5 and 10 years of age, and 3/13 (23.1%) between 10 and 16 years of age (Table 1).

The GCS at the admission in the emergency department was 14 to 15 in 11/13 (84.6%) cases, 9 to 13 GCS in 1/13 (7.7%) cases, and below 8 GCS in 1/13 (7.7%) cases (Table 2). The average GCS at the admission among the not operated patients was 13.6.

One out of thirteen (7.7%) conservatively treated children had a unilateral fixed pupil at admission, and a delayed deterioration of the level of consciousness occurred in 1/13 (7.7%) patients (Table 2), in whom the GCS fell from 13 to 10 during the 3rd day of hospitalization as a result of brain edema.

**Fig. 7:**
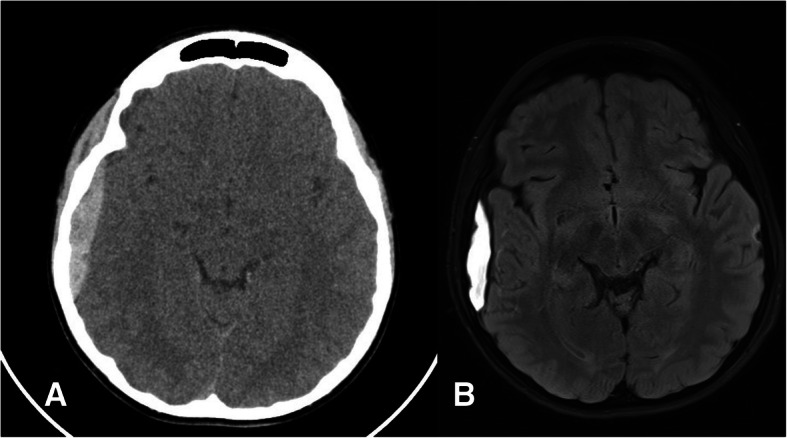
CT scan of an EDH treated conservatively (**a**) and the MRI performed 2 weeks after trauma (**b**) showing a slight reduction of the EDH size

The location of the EDH was temporal in 5/13 (38.5%) cases (Fig. 7), frontotemporal in 4/13 (30.8%) cases, parietotemporal in 2/13 (15.4%) cases, occipital in 1/13 (7.7%) cases, and retroclival in 1/13 (7.7%) cases (Fig. 3).

The mean size of the EDH was 6.8 mm (min. 3 mm, max. 8 mm), and a midline shift was never present (Table 3).

A skull fracture was noticed in 10/13 (76.9%) cases, none of which was dislocated (Table 4). Among the operated children, the EDH was associated with another intracranial lesion in 5/13 (38.4%) cases. There was a contusion in 2/13 (15.4%) cases, a SAH in 2/13 (15.4%) cases, a SDH in 1/13 (7.7%) cases, and brain edema in 1/13 (7.7%) cases at the initial CT scan, while the control imaging revealed brain edema in 2/13 (15.4%) other cases.

The average length of hospitalization in the ICU among the conservatively treated children was 0.8 days, and the time of hospitalization was 7.7 days (min. 1 day, max. 21 days) (Table 5).

The outcome at the discharge from the hospital was (Table 6) as follows: mRS 0 in 10/13 (76.9%) cases and mRS 1–2 in 3/13 (23.1%) cases (2 cases of mild hemiparesis and 1 case of bilateral 6th nerve palsy). Six months after the trauma, 2 children with hemiparesis recovered completely and the outcome was as follows: mRS 0 in 12/13 (92.3%) cases and mRS 1–2 in 1/13 (7.7%) cases, as a result of the 6th nerve palsy, which only partially improved.

## Discussion

Although EDH in children is a potentially life-threatening condition, it can be managed with excellent outcomes as a consequence of access to modern imaging modalities to neurosurgical and ICU treatment. Falls are the most frequent cause of EDH, even when the fall occurs from a height lower than 1 m [7]. EDH are most commonly encountered in familiar environment [8-11] and often after a relatively minor head trauma. Many of these children are alert at admission and present with irritability, headache, nausea, or vomiting, but with no focal neurological deficits [12]. These subtle presenting symptoms make EDH sometimes difficult to diagnose.

There is a male predominance reported in the literature [3, 9, 13] that was confirmed also in our findings, where 83.3% of patients were boys.

The injury of the middle meningeal artery, which is a common source of bleeding in EDH, is often caused by a skull fracture, which is therefore a common finding in EDH and was present in 70% of our cases. In other series, the observations were similar and the range of skull fractures was reported between 48 and 90% [8, 9, 11, 14].

The arterial origin of EDH in older children and young adults explains also the rare, but typical three-phasic clinical manifestation of EDH, in which an initial loss of consciousness is followed by a lucidity interval and finally by a second loss of consciousness, with frequent unilateral mydriasis. Pasaoglu et al. reported this lucidity interval in 32% of their pediatric patients [11], while we observed it in 3/30 (10%) cases. On the contrary to this early progression of symptoms, a clinical deterioration that occurs more than 3 days after trauma is defined as late [7]. While an early deterioration is directly caused by an EDH enlargement, the delayed deterioration is related mostly to secondary brain edema and to a prolonged mass effect. A delayed deterioration was observed in 2/30 (6.7%) cases in our series, and 1 of these patients needed a surgical evacuation.

Whether a surgical or a conservative treatment of EDH is more appropriate remains a controversial topic, and there are no widely accepted protocols for the management of these patients. Factors for surgical treatment include volume > 30 mL, thickness > 15–18 mm, and shift > 4–5 mm [15, 16]. On the other hand, a conservative treatment is indicated in case of thickness < 1 cm, antero-posterior diameter < 3 cm, no midline shift, and absence of neurologic deficits [9, 10, 17-19]. Nevertheless, Balmer et al. described a series of children with EDH larger than 1 cm treated conservatively and showed that size alone does not represent a pure indication for surgical treatment [7]. Our surgical indications depended mainly on EDH thickness, on GCS, and on the presence of midline shift and of a dislocated fracture.

Beyond these indications, subjectivity of the attending surgeon still plays a major role and there is a zone of uncertainty in the process of decision making, particularly in a group of children presenting with unspecific clinical signs and symptoms, EDH thickness between 8 and 12 mm, no shift, and GCS between 12 and 14. These cases have been defined by Bejjani et al. as intermediate-size EDH, and for these cases, the authors suggested a careful and individualized clinical management [15].

Based on the statistical analysis of our series, no significant relationship was found between the type of treatment and the final outcome (*p* = 0,231). On the basis of the low risk of neurosurgical treatment, we can assume that surgical evacuation is safe also for the intermediate-size EDH. On the opposite, the decision to follow-up closely these patients must consider the possibility of a late deterioration [7], which appeared in 2/30 (6.7%) cases in our series. Therefore, conservative management can only be performed in an ICU environment and in hospitals with neurosurgical attendance, with the possibility to perform a craniotomy at any time [7, 10].

Poor prognostic factors described so far for EDH include bradycardia [17, 20], seizures, focal neurological deficits [14], age [21], and mydriasis [8, 14, 17, 20]. The presence of associated brain contusions was not directly related to bad outcome [9]. In our series, the only prognostic factor significantly related to the presence of neurological deficits at 6 months (mRS 1–5) was a GCS below 8 at admission. Similar findings have already been described by other authors [14, 17, 20, 22].

The mortality of EDH in literature ranges from 0 to 12% [2, 9, 14, 17, 20, 22, 23]. In our series, no mortality was documented, which might be explained by the advanced contemporary pediatric trauma care and urgent surgery in patients with clinical signs of uncal herniation.

Operative treatment did not bring any morbidity or complications in our series, which is consistent with other reports [9, 24]. Beyond this, the final outcome was slightly worse in the operated group (76.4% of mRS 0) compared to the conservatively treated group (92.3% of mRS 0). This difference was not statistically significant and might reflect the worse initial neurological conditions that were more frequent in the operated group. Overall, 83.3% of all EDH had an excellent outcome (mRS 0) 6 months after the discharge. These results are similar to other recent reports of children treated both surgically and conservatively [7, 9].

## Conclusions

Our study shows that pediatric EDH can be managed with good outcome, independently from the initial GCS score, from the presence of focal neurological deficits, and from the EDH thickness. The only poor prognostic factor is a low GCS at admission.

## Data Availability

Data sharing is not applicable to this article as no datasets were generated or analyzed during the current study.

## Abbreviations

CVI: Cerebrovascular insult
EDH: Epidural hematoma
GCS: Glasgow Coma Scale
ICP: Intracranial pressure
ICU: Intensive care unit
mRS: Modified Rankin Scale
SAH: Subarachnoid hemorrhage
SDH: Subdural hematoma
